# UKEV forum 2024: The UK Society for Extracellular Vesicle annual meeting - bridging innovation and impact

**DOI:** 10.20517/evcna.2025.62

**Published:** 2025-08-21

**Authors:** Hannah K. Jackson, Naveed Akbar, Nick Peake, Ryan C. Pink, Charlotte Lawson

**Affiliations:** ^1^Pain Centre Versus Arthritis, University of Nottingham, Medical School, Queen’s Medical Centre, Nottingham NG7 2UH, UK.; ^2^School of Life Sciences, Medical School, Queen’s Medical Centre, Nottingham NG7 2UH, UK.; ^3^Division of Cardiovascular Medicine, Radcliffe Department of Medicine, University of Oxford, Oxford OX3 9DU, UK.; ^4^Biomolecular Sciences Research Centre, Sheffield Hallam University, Sheffield S1 1WB, UK.; ^5^Department of Biological and Medical Science, Faculty of Health and Life Sciences, Oxford Brookes University, Oxford OX3 0BP, UK.; ^6^School of Pharmacy and Biomedical Sciences, University of Central Lancashire, Preston PR1 2HE, UK.

**Keywords:** Extracellular vesicles, biomarkers, EV isolation, dementia, lung disease, gene therapy, UKEV, translational medicine

## Abstract

In December 2024, the United Kingdom Society for Extracellular Vesicles (UKEV) held its annual forum in Newcastle upon Tyne, marking 11 years since its founding. UKEV Forum 2024 brought together over 230 participants from the UK and abroad under the theme “Bridging Innovation and Impact”. The meeting emphasised translational science, regulatory foresight, methodological rigour, and cross-sector collaboration, reflecting the maturation of EV research towards clinical relevance. Hosted at Newcastle University, the event included plenary lectures, oral and lightning talks, poster sessions, and an Early Career Researcher (ECR) Day. Scientific discussions spanned EV biomarker discovery, mechanistic studies, tissue-derived EVs, and novel analytical tools such as cryo-TEM, electrochemical sensors, and DNA-PAINT microscopy. The forum showcased emerging topics in EV isolation, reproducibility, therapeutic development, and regulatory integration, drawing on diverse expertise across academia, biotech, and clinical sciences. Generous industry sponsorship and inclusive programming made UKEV 2024 a landmark event that reinforced the UK’s leadership in EV research.

## EARLY CAREER RESEARCHER DAY: FOSTERING THE NEXT GENERATION

The forum officially commenced with a dedicated Early Career Researcher (ECR) Day on December 16th, designed to support and inspire the next generation of extracellular vesicles (EV) scientists. Attended by over 70 postgraduate students and postdoctoral researchers, the ECR Day included sessions on career trajectories, academic-industry collaboration, and research integrity. Talks from Dr. Christopher Stewart and industry representatives such as Caitlin Richardson (Ramarketing) and Martina Miotto (CellRev) offered valuable perspectives on transitioning between academia and biotech. Stacey Wagstaff’s presentation on artificial intelligence (AI) and research integrity initiated a thoughtful discussion on the responsible use of emerging generative technologies in biological research.

Interactive workshops explored burnout prevention, work-life balance, and the benefits of collaborative networks, including the Midlands Innovation EV network. A key takeaway was the importance of mentorship and open scientific dialogue, and participants voiced appreciation for the networking drinks event held at Northern Stage, where informal conversations and socialising have led to potential collaborations.

ECR attendees emphasised the value of mentorship and practical advice, an overarching theme that resonated throughout the forum. Feedback suggested that the ECR day helped demystify academic and non-academic career paths while reinforcing community and inclusivity within the EV field.

## PLENARY LECTURES AND THEMATIC SESSIONS

The scientific programme began with a keynote address by Prof. An Hendrix (Ghent University), whose talk addressed the critical influence of EV isolation methods on downstream applications. Using comparative studies involving OptiPrep density gradient (ODG), differential ultracentrifugation (dUC), and size exclusion chromatography (SEC), Hendrix demonstrated how RNA profiles and immunological outcomes depend on preparation methodology. Emphasis was placed on the value of the EV-TRACK knowledgebase and EVQC toolkit in enhancing reproducibility across the field. Practical advice on storage, sample handling, and removal of lipoproteins further anchored the talk in translational relevance.

Day 2 featured a compelling keynote by Prof. Ken Witwer (Johns Hopkins University), who addressed the translational pipeline from discovery to therapy. Drawing on lessons from the failure of EV-based clinical trials in oncology, Witwer underscored the importance of bridging academic and industrial priorities and called for regulatory foresight, model standardisation, and shared preclinical resources. His metaphor of “a city of bridges” served as a fitting reflection of the host city’s legacy and the Society’s vision. He reflected on the field’s lessons, from failed oncology trials to EV biodistribution studies in rodents and macaques, highlighting immune clearance, dosing concerns, and the need for large animal models. Witwer urged researchers to focus on EV surface biology, ligand-receptor interactions, and immunogenicity. He challenged the field to confront gaps in multi-omic integration, engage regulators proactively, and not shy away from complexity. Importantly, he encouraged humility in addressing the unknowns, what he called “bridging the data gaps”.

The final keynote came from Prof. Rossella Crescitelli (University of Gothenburg, Sweden), who showcased the emerging frontier of tissue-derived EVs (tEVs). Her team’s work demonstrated the stability and diagnostic relevance of tEVs in cancer, including mitochondrial protein signatures like COX6C and MTC02 as plasma biomarkers in melanoma. She highlighted rigorous comparisons between fresh and frozen tissue sources, validated her isolation methods via 3D electron tomography, and outlined new immunotherapy strategies combining tEVs and synthetic vesicles to reduce tumour burden in mice. Prof. Crescitelli also discussed ongoing efforts to formalise tEV protocols through the ISEV Solid Tissue Task Force.

## ORAL PRESENTATIONS: MECHANISTIC INSIGHT AND CLINICAL POTENTIAL

The oral sessions at the United Kingdom Society for Extracellular Vesicles (UKEV) Forum 2024 showcased high-impact research that advanced mechanistic understanding and translational application of EVs across disease contexts. Fatma Busra Isik’s work stood out for identifying microglial EV biomarkers for Dementia with Lewy Bodies, using TMEM119-based enrichment and RNA profiling to differentiate Alzheimer’s disease subtypes with a blood-based signature. This enrichment approach provides a promising strategy for blood-based differential diagnosis of neurodegenerative diseases.

Kestecher Brachyahu Meir’s mouse model work explored CD63+ EV dynamics in hypercholesterolemia, linking EV profiles to cardiac function under dietary stress. His proposal to test LOX1 blockade as a means to probe EV uptake mechanisms bridged mechanistic insight and translational aspiration. Meir proposed future studies involving LOX1 blockade to assess EV uptake and suggested that EV profiles may serve as more sensitive indicators of cardiovascular risk than cholesterol levels alone.

Drs. Rahul Mahida and Katie Spencer from the University of Birmingham delivered a complementary set of talks focused on EV-driven macrophage dysfunction in fibrotic and inflammatory lung conditions. Employing co-culture systems, lipidomics, and transcriptomics, their data showed miR-15a and miR-26b impair efferocytosis in alveolar macrophages and correlate with poor patient outcomes in acute respiratory distress syndrome (ARDS) and idiopathic pulmonary fibrosis (IPF).

Morganne Wilbourne focused on placental EV miRNAs in preeclampsia (PE). By comparing ncRNA profiles of placental tissue to those in small and medium/large EVs, she found that PE-associated EVs carried enriched miRNAs linked to immune dysfunction and sterile inflammation. This work underscores the potential of EV cargo to reflect placental pathology and inform maternal-foetal health interventions.

Collectively, these talks demonstrated the multifaceted role of EVs in neurodegeneration, cardiovascular, and pulmonary diseases, revealing their utility not only as biomarkers but also as targets for therapeutic intervention.

## POSTER HIGHLIGHTS AND THEMATIC TRENDS

With over 80 posters on display, the forum showcased breadth and creativity across the EV research spectrum. Posters ranged from molecular profiling of urinary EVs and Parkinson’s disease diagnostics to regenerative applications using mesenchymal stromal cell-derived EVs. Noteworthy contributions included Dr. Abigail Byford’s work on maternal EVs in gestational diabetes, Miss Georgina Thompson’s study of cardiac fibroblast-derived EVs in hypertrophic cardiomyopathy, and Dr. Elizabeth Dellar’s optimisation of cerebral spinal fluid (CSF) EV immunocapture.

Lucinda Cruddas explored the potential role of small EVs in chronic diabetic foot ulcers, reporting size-dependent EV secretion patterns in fibroblasts under hyperglycaemic and hypoxic stress. These findings contributed to a broader theme: EV heterogeneity and how stress conditions influence cargo sorting.

Several posters embraced multi-omic integration, and others employed novel visualisation techniques, such as DNA-PAINT microscopy, Raman spectroscopy, or nano-flow cytometry, reflecting the increasing technical diversity of the field.

## LIGHTNING TALKS AND EMERGING TOPICS

The lightning talk sessions provided an energetic platform for early-stage researchers and postdoctoral fellows to present innovative findings across a wide spectrum of EV research. Dr. Saskia Bakker’s application of cryogenic transmission electron microscopy (cryo-TEM) to dairy-derived EVs, Prof. Nicholas Turner’s electrochemical profiling of cancer cell EVs, and Yağmur Yıldızhan’s mechanistic insights into exosome tethering all illustrated creative, cross-cutting applications of EV science.

Additional highlights included Himadri Devvanshi’s exploration of placental EVs in preterm birth, Soraya Williams’ profiling of mesenchymal EVs in regenerative dermatology, and Tim Williams’ demonstration of antimicrobial proteins in urinary EV subtypes. These diverse applications underscored the continued evolution of EVs from experimental curiosity to biological necessity.

## PANEL DISCUSSION AND AGM: LOOKING AHEAD

A highlight of UKEV Forum 2024 was the plenary panel discussion featuring Profs. Rossella Crescitelli, An Hendrix, and Ken Witwer, who led a forward-looking conversation on the future of EV research in the UK and globally. The panel tackled critical themes such as regulatory frameworks, clinical translation, data reproducibility, and infrastructure development. Witwer reiterated the importance of aligning academic output with regulatory expectations, calling for early engagement with agencies like the U.S. Food and Drug Administration (FDA) and Medicines and Healthcare products Regulatory Agency (UK) (MHRA). He encouraged researchers to fully characterise EV preparations and document safety, including potential immunogenicity and the presence of DNA, viruses, or endotoxins.

Prof. Crescitelli proposed the retrospective use of biobanked tissues for EV studies, noting that her group successfully isolated EVs from both fresh and frozen melanoma samples with comparable quality. She advocated for a global EV tissue mapping initiative, akin to the Human Protein Atlas, to systematically catalogue tEV profiles across organs and disease states.

The discussion explored whether the field is ready for multi-omic profiling of small EV samples. While panellists agreed on its necessity, they noted the financial and technical hurdles to deep sequencing and proteomics on low-input material. Emerging single-EV methods, such as Raman spectroscopy, DNA-PAINT, and nano-flow cytometry, were seen as promising complements, though consensus emerged that no universal EV marker has yet been identified. From the floor, community questions highlighted the gap between academia and industry, particularly in therapeutic applications. Panellists stressed the need for open communication, early partnerships, and realistic expectations on both sides. Academia excels at exploration and mechanism discovery, while industry is equipped for translation and scalability.

At the annual general meeting (AGM), the Society reaffirmed its commitment to equity and training, with votes supporting the establishment of a new equality, diversity, and inclusion (EDI) working group, a mentoring programme for junior scientists, and the expansion of regional meetings to increase accessibility. Members also discussed opportunities for UKEV to influence policy through white papers and cross-sector alliances.

## INTERNATIONAL AND INTERDISCIPLINARY PARTICIPATION

This year’s forum included contributions from researchers based in Italy, India, the United States, and several European (EU) countries, reflecting UKEV’s widening international reach. Oral and poster presenters brought expertise from diverse disciplines including bioengineering, veterinary science, reproductive health, and synthetic biology. Such interdisciplinary input reinforced the notion that EV science is not just a subdiscipline but a foundational component across biomedical research.

ISEV board members in attendance commented on the UK’s leadership in the field and expressed interest in deepening ties between UKEV and ISEV through shared initiatives. The possibility of educational workshops and a regional ISEV meeting in the UK were highlighted as potential avenues for collaboration between ISEV and UKEV, and follow-up discussions are expected in the coming months.

## COMMUNITY BUILDING AND INCLUSION

Beyond the science, UKEV Forum 2024 reaffirmed the Society’s commitment to community. Delegates appreciated the ample networking time, inclusive programming, and social events. The Civic Centre dinner, complete with local cuisine and entertainment, was a highlight for many attendees. Several first-time attendees noted the welcoming atmosphere and accessibility of the sessions.

Importantly, UKEV’s steps towards equity and inclusion were visible throughout the event. All-gender restrooms, quiet rooms, and accessibility guidance were made available, and diverse speakers were represented across sessions. The AGM included a proposal for establishing an EDI working group within the Society to help shape policy and future initiatives.

## BRIDGING TO THE FUTURE: UKEV’S NEXT CHAPTER

Looking forward, UKEV aims to play a pivotal role in aligning UK EV research with global efforts. The Society is actively exploring opportunities for a UK-hosted regional ISEV conference and the creation of a centralised EV resource repository for training and method sharing. Plans are also underway to launch a “UKEV Excellence in Outreach” prize, recognising efforts to bring EV science to the public and patient audience.

As EV research accelerates into therapeutic realms, UKEV Forum 2024 highlighted that collaboration, clarity, and community remain core to the Society’s mission. Whether through mentoring young investigators, embracing open science, or championing diversity, the UKEV community is well-positioned to lead with both scientific excellence and social responsibility.

## LOOKING AHEAD TO UKEV FORUM 2025

We are pleased to confirm that the UKEV Forum 2025 will be held in Oxford, at the Andrew Wiles Building, Oxford Mathematics, University of Oxford. The conference will be hosted by the Oxosome Team in collaboration with the UKEV Board. The meeting will commence with the ECR Day on Monday, 15th December, followed by the main Forum on Tuesday, 16th, and Wednesday, 17th December. Following the success of UKEV Forum 2024, next year’s meeting will expand to three days of engaging and impactful EV science. The programme will feature international and UK-based keynote speakers presenting innovations in extracellular vesicle research, alongside selected talks, poster sessions, and lightning presentations from the UK research community.

Key thematic areas will include EV detection and quantification, basic EV biology, biomarker discovery, EV roles in health and disease, and EV-based therapeutics. UKEV Forum 2025 aims to further enhance community building, cross-sector collaboration, and international engagement. We look forward to welcoming delegates to Oxford for another inspiring chapter in UK EV research.

## DECLARATIONS

### Copyright

© The Author(s) 2025.

